# Carbamoylating Activity Associated with the Activation of the Antitumor Agent Laromustine Inhibits Angiogenesis by Inducing ASK1-Dependent Endothelial Cell Death

**DOI:** 10.1371/journal.pone.0103224

**Published:** 2014-07-28

**Authors:** Weidong Ji, Mei Yang, Alexandra Praggastis, Yonghao Li, Huanjiao Jenny Zhou, Yun He, Roxanne Ghazvinian, Dylan J. Cincotta, Kevin P. Rice, Wang Min

**Affiliations:** 1 No.1 Affiliated Hospital, Sun Yat-sen University, Guangzhou, China; 2 Breast Disease Center, Guangdong Women and Children Hospital of Guangzhou Medical University, Guangzhou, P.R. China; 3 Department of Chemistry, Colby College, Waterville, Maine, United States of America; 4 State Key Laboratory of Ophthalmology, Zhongshan Ophthalmic Center, Sun Yat-sen University, Guangzhou, China; 5 Interdepartmental Program in Vascular Biology and Therapeutics, Department of Pathology, Yale University School of Medicine, New Haven, Connecticut, United States of America; UAE University, Faculty of Medicine & Health Sciences, United Arab Emirates

## Abstract

The anticancer agent 1,2-bis(methylsulfonyl)-1-(2-chloroethyl)-2-[(methylamino)carbonyl]hydrazine (laromustine), upon decomposition in situ, yields methyl isocyanate and the chloroethylating species 1,2-bis(methylsulfonyl)-1-(2-chloroethyl)hydrazine (90CE). 90CE has been shown to kill tumor cells via a proposed mechanism that involves interstrand DNA cross-linking. However, the role of methyl isocyanate in the antineoplastic function of laromustine has not been delineated. Herein, we show that 1,2-bis(methylsulfonyl)-1-[(methylamino)carbonyl]hydrazine (101MDCE), an analog of laromustine that generates only methyl isocyanate, activates ASK1-JNK/p38 signaling in endothelial cells (EC). We have previously shown that ASK1 forms a complex with reduced thioredoxin (Trx1) in resting EC, and that the Cys residues in ASK1 and Trx1 are critical for their interaction. 101MDCE dissociated ASK1 from Trx1, but not from the phosphoserine-binding inhibitor 14-3-3, in whole cells and in cell lysates, consistent with the known ability of methyl isocyanate to carbamoylate free thiol groups of proteins. 101MDCE had no effect on the kinase activity of purified ASK1, JNK, or the catalytic activity of Trx1. However, 101MDCE, but not 90CE, significantly decreased the activity of Trx reductase-1 (TrxR1). We conclude that methyl isocyanate induces dissociation of ASK1 from Trx1 either directly by carbamoylating the critical Cys groups in the ASK1-Trx1 complex or indirectly by inhibiting TrxR1. Furthermore, 101MDCE (but not 90CE) induced EC death through a non-apoptotic (necroptotic) pathway leading to inhibition of angiogenesis in vitro. Our study has identified methyl isocyanates may contribute to the anticancer activity in part by interfering with tumor angiogenesis.

## Introduction

The prodrug Laromustine [1,2-bis(methylsulfonyl)-1-(2-chloroethyl)-2-[(methylamino)carbonyl]hydrazine] yields two reactive electrophiles, methyl isocyanate and 90CE, upon base-catalyzed activation in situ, which carbamoylate and chloroethylate, respectively, receptive nucleophiles in the cell [1], [2]. The chloroethylation of the O6 position of guanine in DNA is believed to be the major cytotoxic lesion, resulting in an interstrand DNA crosslink that is difficult for the cell to repair [3], [4]. The other reactive component, methyl isocyanate, preferentially carbamoylates sulfhydryl groups, but also attacks amine and hydroxyl groups. 101MDCE, an analog of Laromustine that lacks chlorethylating activity while retaining carbamoylating activity, not only is cytotoxic towards cultured neoplastic cells by itself, but also produces synergistic cell kill with 90CE [3], [4]. One likely target of the carbamoylating activity of Laromustine is O6-alkylguanine-DNA alkyltranferase (AGT), a protein which when overexpressed, renders neoplastic cells resistant to alkylating agents that target the O6 position of guanine in DNA. The precise mechanism by which methyl isocyanate contributes to the antineoplastic activity of Laromustine is not fully understood.

The antitumor DNA-alkylating agent 1,3-bis(2-chloroethyl)-1-nitrosourea (BCNU; Carmustine) also generates an alkyl isocyanate upon decomposition [5]–[7]. However, rather than methyl isocyanate, BCNU produces 2-chloroethyl isocyanate [6]. Although both methyl and 2-chloroethyl isocyanates can readily carbamoylate sulfhydryl groups, there are significant functional differences between these reactive species in cells. One example of such differences involves the enzyme glutathione reductase (GR). BCNU inhibits cellular GR by up to 90% at pharmacological doses, a phenomenon implicated as a cause of the pulmonary toxicity often seen in high-dose BCNU-treated animals and human cancer patients [8]. We have recently demonstrated that Laromustine does not produce similar inhibition of cellular GR activity in human erythrocytes and L1210 murine leukemia cells, despite BCNU and Laromustine being equally potent inhibitors of the purified human enzyme (IC_50_ values of 55.5 µM and 54.6 µM, respectively) [9]. Given the known significance of the contribution of the methyl isocyanate towards the therapeutic efficacy of Laromustine and the observed differences between Laromustine and BCNU in the inhibition of cellular GR, it is likely that the critical target(s) of methyl isocyanate has not been fully revealed.

The thioredoxin system, which involves thioreodoxin (Trx), Trx reductase (TrxR), and Trx peroxidase, is another endogenous antioxidant system. Trx contains two redox-active cysteine residues in its catalytic center with the consensus amino acid sequence –cys-gly-pro-cys. Trx can exist either in a reduced dithiol form or in an oxidized form and participates in redox reactions by reversible oxidation of its active center dithiol to disulfide and catalyzes dithio-disulfide exchange reactions involving many thiol-dependent processes [10]–[12]. TrxR converts oxidized Trx to its reduced form. The Trx-TrxR system has multiple functions in the cell, including regulation of cell growth, apoptosis, and activation processes [10]–[12]. Trx can prevent cellular apoptosis by scavenging reactive oxygen species (ROS), thereby providing protection from oxidative stress. It also acts anti-apoptotically by regulating the activities of transcription factors such as NF-kB and AP-1, and by directly binding and inhibiting the activity of the pro-apoptotic protein apoptosis signal-regulating kinase 1 (ASK1) [13]–[16].

ASK1, a member of the MAP3K family activating MAP2K–JNK/p38 cascades, can be activated in response to various stress stimuli, including pro-inflammatory cytokines, oxidative stress, ER stress and genotoxic reagents [17], [18]. Although ASK1 functions in the proliferation, differentiation, and survival of various cell types, its role in cell death has been most extensively studied [18]. Studies in overexpression systems and from ASK1 knockout mice have shown that ASK1 is a critical mediator in tumor necrosis factor (TNF), ROS, and stress-induced cell death [19], [20]. ASK1 is reported to induce apoptosis by triggering a mitochondria-dependent pathway [16], [21]. ASK1 also induces non-apoptotic cell death [22]. ASK1 is a 170 kD protein that is composed of an inhibitory N-terminal domain, an internal kinase domain, and a C-terminal regulatory domain. Several intracellular proteins bind to different domains of ASK1, keeping it in an inactive state in resting cells. The redox sensor protein Trx binds to the N-terminal domain of ASK1, while glutaredoxin binds to the C-terminal domain, in each case, inhibiting ASK1 kinase activity [23], [24]. Phosphoserine-binding protein 14-3-3 associates with ASK1 via the pSer967 site of ASK1 and inhibits ASK1-induced apoptosis [15], [25]–[28]. Stress stimuli activate ASK1, in part by dissociating ASK1 from its inhibitors. Activation of ASK1 leads to death in many cell types, including tumor cells and EC. Thus, ASK1 is a central target of many cellular survival factors, including Raf-1, Akt, and hsp90 [29]–[31]. It is conceivable that ASK1 is a potential therapeutic target for anticancer drugs. Indeed, paclitaxel, a microtubule-polymerizing agent used in cancer chemotherapy, induces activation of ASK1 in human cells [32]. In addition, we have recently shown that the hsp90 inhibitor, 17-AAG, a preclinical drug for cancer therapy, also induces ASK1-JNK signaling in EC [31]. Furthermore, there has been some preclinical investigation of co-administration of BCNU and known anti-angiogenic agents [33].

In the present study, we have shown that Laromustine and 101MDCE, but not 90CE, induce ASK1-JNK activation in EC. 101MDCE dissociates ASK1 from Trx, but not from the phosphoserine-binding inhibitor 14-3-3. We further show that none of the methylsulfonyl hydrazines have any effect on the kinase activity of purified ASK1, JNK, or the catalytic activity of Trx. However, agents with carbamoylating activity significantly inhibit TrxR1 activity. Moreover, 101MDCE, but not 90CE, induces EC death through a non-apoptotic pathway and inhibits EC tube formation. These findings suggest that methyl isocyanate inhibits angiogenesis by inducing ASK1-JNK-dependent EC death.

## Results

**Figure 1:**
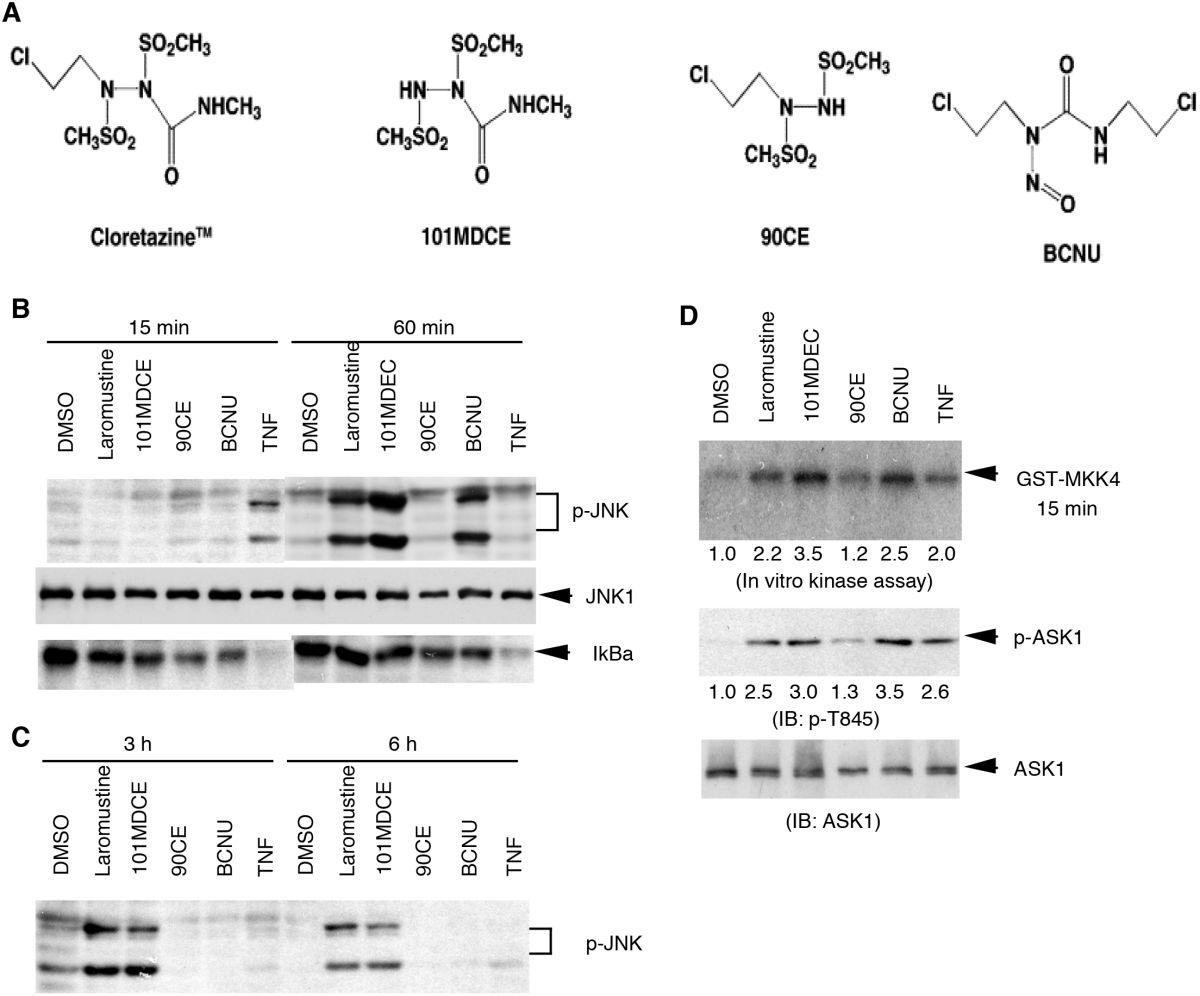
Effects of 101MDCE on the activation ASK1-JNK pathways. A. Chemical structures of sulfonylhydrazine prodrugs and BCNU. B–C. BAEC were treated with Laromustine, 101MDCE, 90CE, or BCNU (200 µM each) for the indicated times (0, 15 min, 1, 3 and 6 h). Phosphorylation of JNK and degradation of IκBα were determined by Western blotting with phospho-JNK and anti-IκBα antibody, respectively. D. ASK1 activation was determined by an in vitro kinase assay using GST-MKK4 as a substrate (top panel) or by Western blotting with a phospho-specific antibody (pThr-845) (bottom panel). ASK1 activity and p-ASK1 are quantified by taking DMSO as 1.0. All experiments were repeated three times and representative blots are shown.

Alkyl isocyanate-generating agents activate ASK1-JNK pathway in EC. To determine the mechanism by which methyl isocyanate derived from Laromustine contributes to anticancer activity, we examined signaling pathways activated by 101MDCE in EC. BAEC were treated with Laromustine, 101MDCE, BCNU, or 90CE (Fig. 1A) (200 µM each) for the indicated times (0, 15, 30 min, 1, 3 and 6 h). JNK/p38 and NF-κB, two pathways critical for EC death and survival, were examined. Activation of JNK/p38 was determined by Western blotting with phospho-specific antibodies. Degradation of IκBα, a marker for NF-κB activation, was determined by Western blotting with anti-IκBα antibody. As a control, 10 ng/ml of TNF was used to induce phosphorylation of JNK and degradation of IκBα, with activation peaking at 15 min and declining by 60 min (Fig. 1b). Laromustine, BCNU, and 101MDCE strongly induced the phosphorylation of JNK, which peaked at 1 h and for Laromustine and 101MDCE, was sustained up to 6 h (Fig. 1B,C). Activation of p38, another member of stress-activated MAPK, showed similar kinetics (data not shown). In contrast, treatment with 90CE, which lacks the carbamoylating activity of methyl isocyanate, did not produce in activation of JNK (Fig.B,C) or p38 (data not shown). Laromustine and 101MDCE failed to induce the degradation of IκBα, while BCNU and 90CE did so only weakly (Fig. 1B,C).

ASK1, a MAP3K upstream of the JNK/p38 cascade, is critical for EC death in response to stress stimuli. To determine the effects of methyl isocyanate on ASK1 activation in EC, BAEC were treated with Laromustine, 101MDCE, 90CE, or BCNU (200 µM each) for 60 min and ASK1 activation was determined by an in vitro kinase assay using GST-MKK4 as a substrate and by Western blotting with a phospho-specific antibody (pThr-845, an autophosphorylation site critical for ASK1 activation). In a manner analogous to JNK activation, Laromustine, 101MDCE, and BCNU strongly activated ASK1 in EC (Fig. 1C). In contrast, treatment with 90CE did not cause the activation of ASK1. These findings suggest that alkyl isocyanates are capable of inducing ASK1-JNK activation.

**Figure 2:**
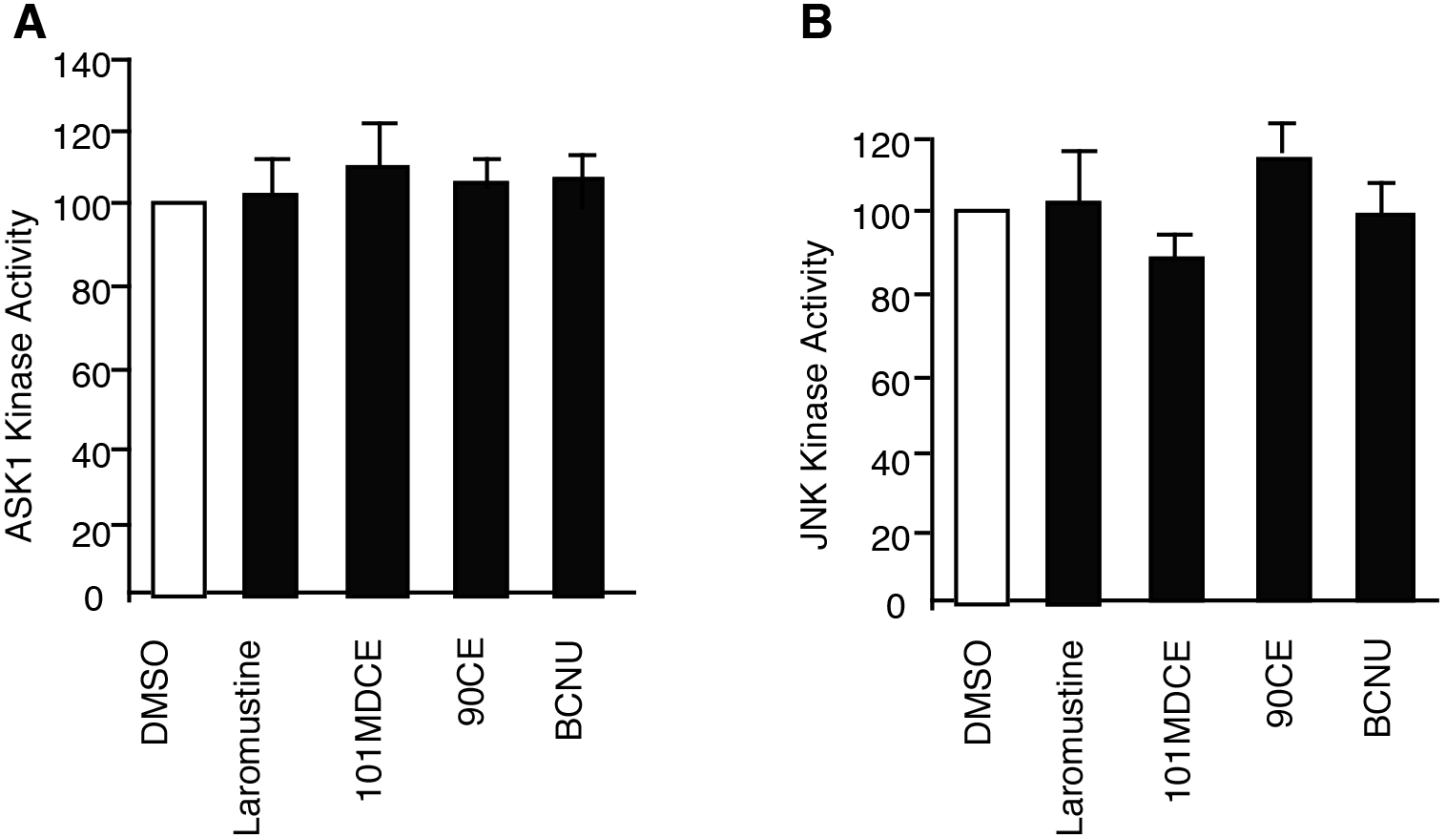
Effects of 101MDCE on the kinase activities of purified ASK1 and JNK1. Purified enzymes were pre-incubated with agents in pH 8 buffer for 1 h. For ASK1 experiments (A), myelin basic protein and ^32^P-labled ATP were added to the enzyme at final concentrations of 20 µM and 100 µM, respectively, then incubated at 25°C for 20 min before separation using SDS-PAGE. For JNK1 experiments (B), biotinylated ATF2 peptide and ^32^P-labled ATP were added to the enzyme at final concentrations of 5 µM and 100 µM, respectively. Reactions were then incubated at 25°C for 20 min before separation using a streptavidin-coated membrane. All data were normalized to the positive and negative controls and reported as triplicate averages of percent activity with standard deviations.

The activities of purified ASK1 and JNK1 are not significantly affected by isocyanate-generating agents. To determine what effect the isocyanate species might have on ASK1 and JNK1, these enzymes were pre-incubated with Laromustine, 101MDCE, 90CE, or BCNU (200 µM each) for 1 h at 25°C in Tris-HCl buffer (pH 8). Such pre-incubations are typical when determining the effects of sulfonylhydrazine and nitrosourea prodrugs on the activity of purified enzymes and allow for base-catalyzed activation of these agents in aqueous solution [3], [4], [9], [34], [35]. The half-lives of Laromustine and BCNU under the stated conditions are approximately 1 h [9], [34]. Thus, in these experiments, only one half-life was allowed to expire before the enzymes were assayed. These conditions were chosen as a compromise between the need to allow for agent activation and the observed instability of the purified enzymes. The half-lives of 90CE and 101MDCE are both under 5 min [3] and thus, are completely activated under these conditions. ASK1 kinase activity was slightly activated by 101MDCE at 200 µM (Fig. 2A). This modest stimulation was also observed with the isocyanate-generating agents Laromustine and BCNU when the concentrations were increased to 400 µM (data not shown). 90CE, which lacks a carbamoylating moiety, had no significant effect on ASK1 activity. Therefore, the demonstrative activation of ASK1 observed when cultured EC were treated with 101MDCE is not the result of a direct action upon the enzyme active site. Similarly, 101MDCE, Laromustine, 90CE, and BCNU had no significant effect on the activity of purified JNK1 in vitro (Fig. 2B).

**Figure 3:**
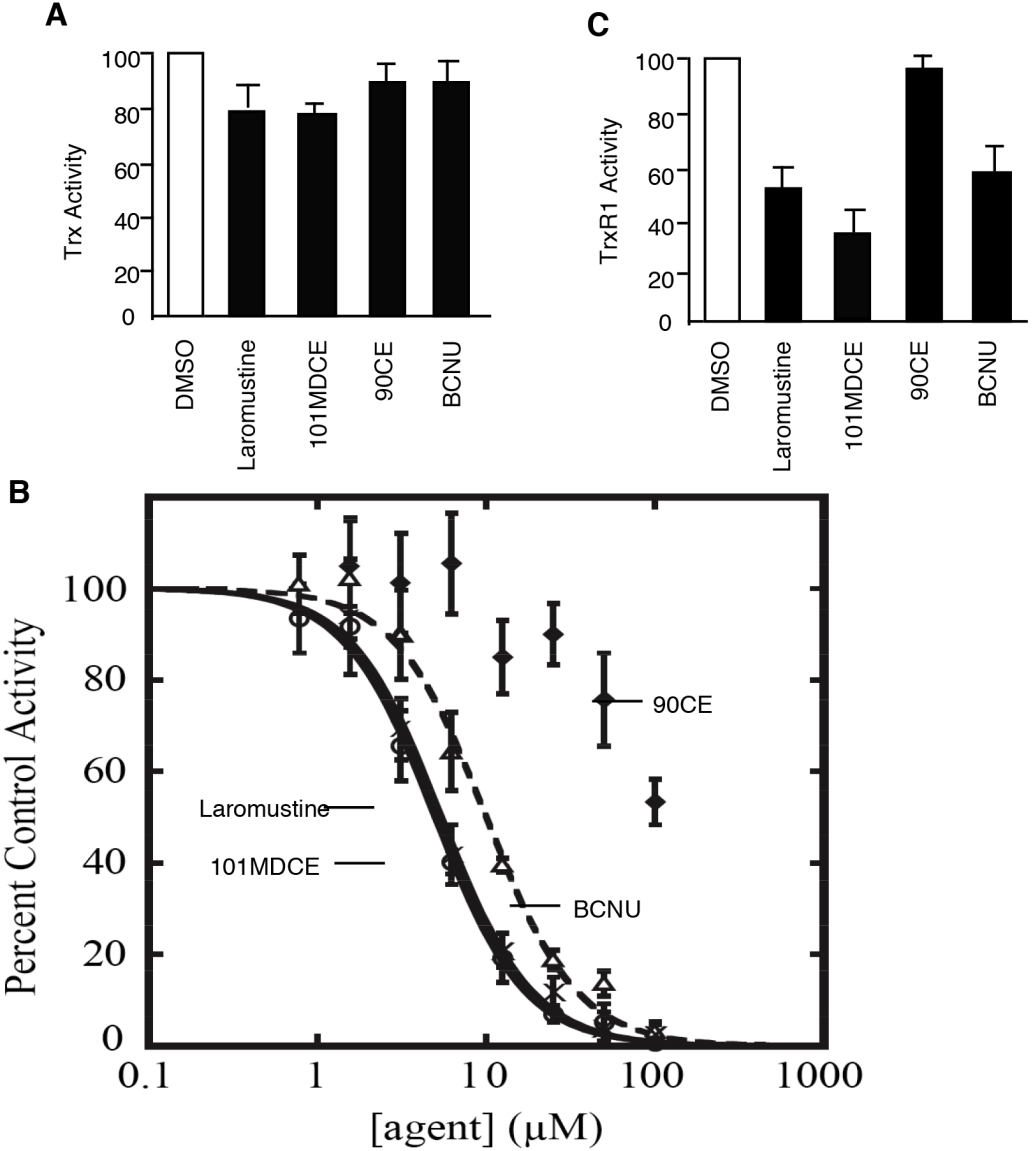
Effects of 101MDCE on the catalytic activities of purified Trx and TrxR1. A. Purified Trx was pre-incubated with agents (10 µM) in pH 8 buffer for 3 h. Insulin and DTT were added to the enzyme at final concentrations of 200 µM and 330 µM, respectively. The mixture was then incubated at 25°C while continuously monitoring absorbance of 650 nm light, which increased as insulin precipitated from solution. All data were normalized to the positive and negative controls and reported as triplicate averages of percent activity with standard deviations. B. Inhibition of purified TrxR1. Laromustine (○), BCNU (Δ), 101MDCE (×), and 90CE (⧫) were serially diluted in DMSO such that equal volumes were added to pre-incubation mixtures of the enzyme. Three h pre-incubations were carried out in the presence of 100 µM NADPH at 25°C in Tris-HCl, pH 8, prior to the addition of the DTNB and NADPH substrates at 5 mM and 300 µM final concentrations, respectively. Reaction velocities were calculated as described in Materials and Methods. C. Inhibition of purified TrxR1 by agents at 10 µM are presented. All values were normalized to a DMSO control and reported as triplicate averages with standard deviations. **, P<0.05; *, P<0.01.

Purified TrxR, but not Trx, is potently inhibited by isocyanate-generating agents. Trx, which inactivates ASK1 by directly binding to the kinase, itself has catalytic redox activity in a wide variety of intracellular molecular processes. To determine the effects of the isocyanate species on Trx, we measured the catalytic activity of Trx1 using insulin as a substrate. The product of this reaction is insoluble at neutral pH and thus can be measured by assessing the turbidity of samples. Treatment with 100 µM of Laromustine, 101MDCE, BCNU, or 90CE only modestly attenuated the activity of Trx1 in this experiment. The methyl isocyanate-generating agents Laromustine and 101MDCE (25% and 21% inhibition, respectively) were slightly more effective than BCNU and 90CE (12% and 9% inhibition, respectively) (Fig. 3A).

TrxR1, the only enzyme known to reduce the oxidized cytosolic form of Trx [36], is a known target of the nitrosourea class of antineoplastic alkylating agents such as BCNU [37]. TrxR1 shares significant homology with GR, which, when purified, is inhibited by both BCNU and Laromustine in vitro [9]. To determine the effects of Laromustine, BCNU, 90CE, and 101MDCE on TrxR1, we performed enzymatic assays using TrxR1, which had been exposed to various concentrations of the agents. Laromustine, 101MDCE, and BCNU inhibited TrxR1 with IC_50_ values of 5.0±1.1, 9.9±3.1, and 5.4±1.4 µM, respectively. These IC_50_ values are approximately an order of magnitude lower than those observed with GR in similar experiments [9]. Consistent with our recent data [35], the inhibition of TrxR1 by 90CE, which is devoid of carbamoylating activity, was much less potent (Fig. 3B), with an IC_50_ value in excess of 100 µM.

**Figure 4:**
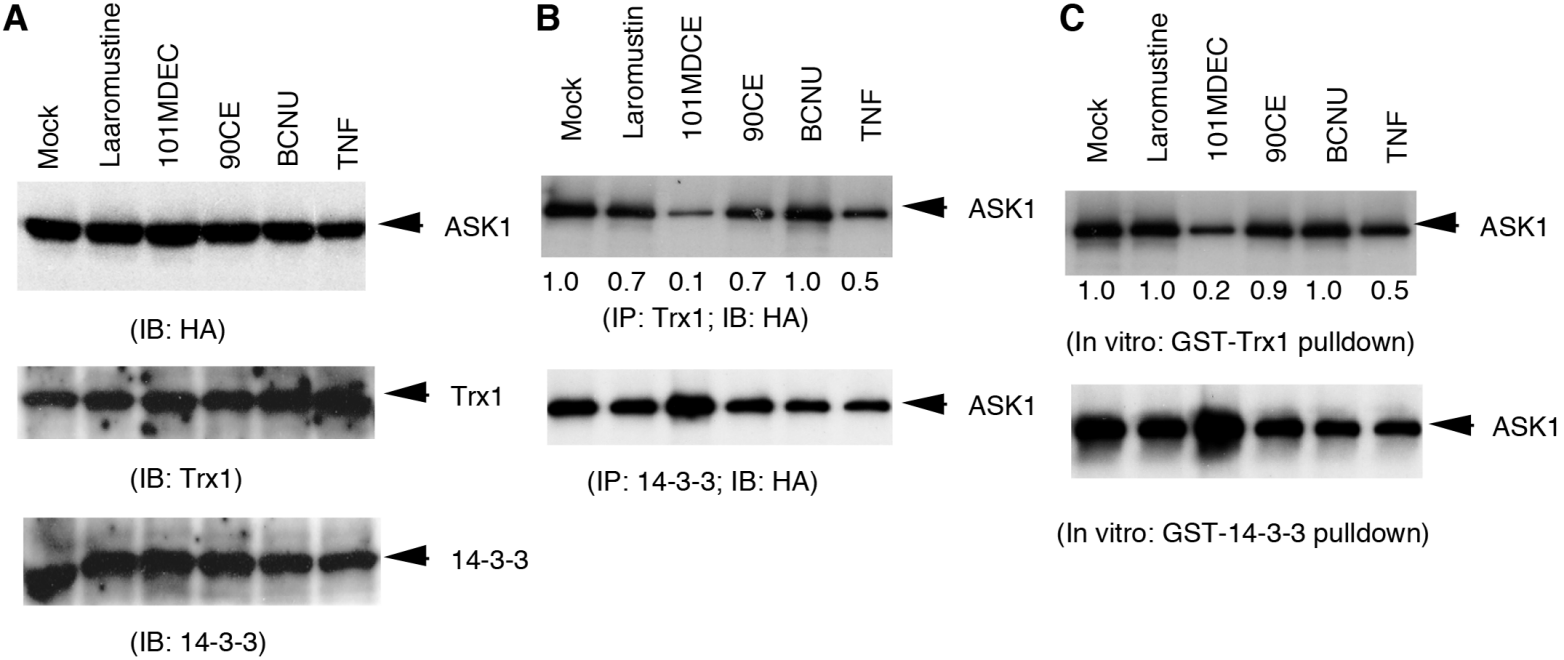
Dissociation of ASK1 from its inhibitor Trx by 101MDCE. BAEC were transfected with HA-tagged ASK followed by treatment with Laromustine, 101MDCE, 90CE, or BCNU (200 µM each) for 1 h. A. Expression of ASK1, Trx and 14-3-3 was determined by Western blot with respective antibodies. B. Association of ASK1 with Trx1 or 14-3-3-3 was determined by immunoprecipitation with anti-Trx1 or anti-14-3-3 followed by Western blotting with anti-HA (for ASK1) antibody. C. Cell lysates containing ASK1 were treated with Laromustine, 101MDCE, 90CE, or BCNU (200 µM each) in vitro for 1 h and association of ASK1 with Trx was determined by an in vitro pulldown assay using GST-Trx or GST-14-3-3 as bait. Bound ASK1 was determined by Western blotting with anti-HA antibody. Relative levels of bound ASK1 are quantified by taking DMSO (mock) as 1.0. All experiments were repeated three times and representative blots are shown.

The methyl carbamoylating agent 101MDCE dissociated ASK1 from its inhibitor Trx1. We have previously shown that ASK1 forms a complex with the reduced form of Trx1 in resting EC, and that the single Cys residue in ASK1 (C250) and C32 or C35 in the cytosolic form of Trx (Trx1) are critical for their interaction [16]. Stimuli such as TNF and H_2_O_2_ dissociate Trx1 from ASK1, presumably by oxidizing the critical Cys residues of Trx1 or ASK1. We reasoned that isocyanates generated from Laromustine and/or BCNU might carbamoylate the thiol groups of Trx1 or ASK1 resulting in Trx1-ASK1 dissociation and ASK1 activation. To test this possibility, we determined the effects of the isocyanate-generating agents on the association of ASK1 with Trx1 (the cytosolic form of Trx). BAEC were transfected with HA-tagged ASK and cells were treated with Laromustine, 101MDCE, 90CE, or BCNU (200 µM each) for 1 h. Association of ASK1 with Trx1 or 14-3-3-3 was determined by immunoprecipitation with anti-Trx1 or anti-14-3-3 followed by Western blotting with anti-HA (for ASK1). 101MDCE strongly induced dissociation of ASK1 from Trx1, while Laromustine and BCNU induced dissociation much more weakly. However, they did not disrupt the ASK1-14-3-3 complex in EC, although 101MDCE may have slightly increased ASK1-14-3-3 association (Fig. 4A). 90CE had no effect on either ASK1-Trx1 or ASK1-14-3-3.

To determine if methyl isocyanate dissociates the ASK1-Trx1 complex outside of the cell, cell lysates containing ASK1 were treated with Laromustine, 101MDCE, 90CE, or BCNU (200 µM each) in vitro for 1 h, and association of ASK1 with Trx1 was determined by immunoprecipitation using anti-Trx1 or anti-14-3-3. Immunoprecipitated ASK1 was detected by Western blotting with anti-HA. Results similar to those using treated cells were observed (data not shown), suggesting that 101MDCE has the ability to dissociate ASK1 from Trx1, but not from 14-3-3. To ascertain if 101MDCE directly modifies ASK1 or Trx1, cell lysates containing ASK1 were treated with Laromustine, 101MDCE, 90CE, or BCNU (200 µM each) for 1 h, and association of ASK1 with Trx1 was determined by an in vitro pulldown assay using GST-Trx1 or GST-14-3-3 as bait. Similarly, 101MDCE strongly decreased the ability of ASK1 to bind to Trx1, but not to 14-3-3 (Fig. 4B). These results suggest that isocyanate species (particularly 101MDCE) might carbamoylate free thiol groups of ASK1.

It is not entirely clear why 101MDCE showed much stronger effects than Laromustine towards the dissociation of ASK1 from Trx1, while sharing similar ability to activate ASK1-JNK signaling. Both agents posess methyl carbamoylating activity when activated in cells. As mentioned above, the half-lives of 90CE and 101MDCE are both under 5 min [3] and therefore, are completely activated in these experiments. However, the half-lives of Laromustine and BCNU under the stated conditions are approximately 1 h [9], [34]. Therefore, the carbamoylating species generated by 101MDCE is provided essentially as a bolus, whereas the isocyanates from Laromustine and BCNU are released only partially and spread out over time. It is possible that the partial activation of Laromustine or BCNU, while sufficient to dissociate all of the cellular ASK1-Trx1 complexes (less abundant), is not sufficient to modify a significant enough fraction of total cellular Trx1 (very abundant). The association of ASK with Trx1 is dynamic, and unmodified Trx1, when treated with Laromustine or BCNU, will re-associate with ASK1 during the immunoprecipitation or the pulldown reaction.

**Figure 5:**
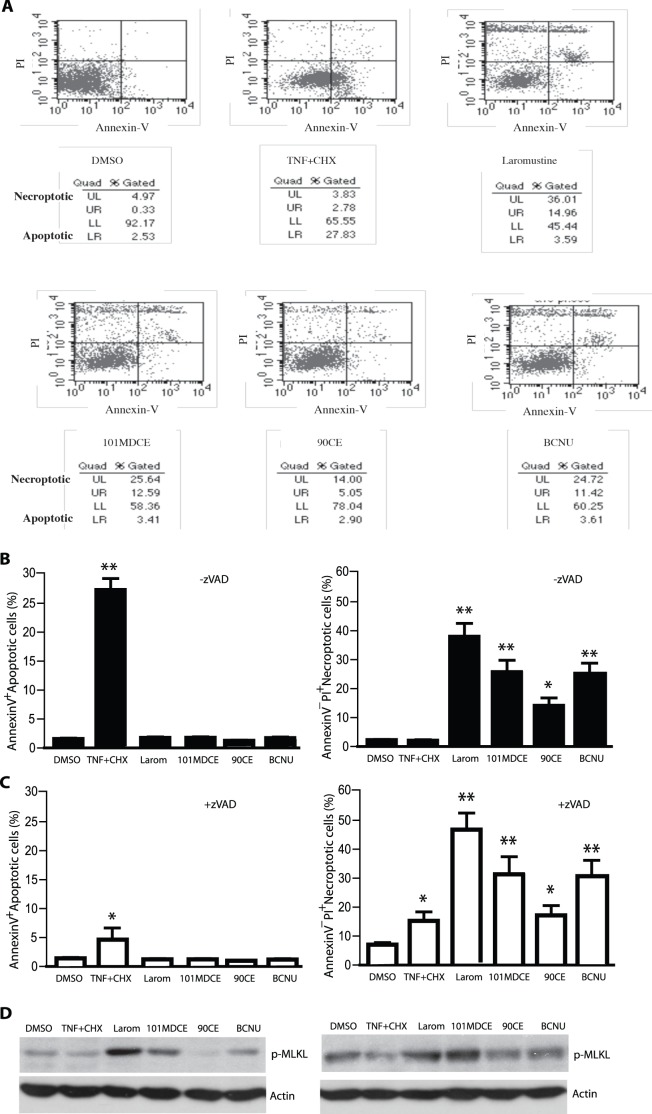
Induction of non-apoptotic cell death in EC by 101MDCE. HUVEC were incubated with agents at 200 µM for 6 h in the absence or presence of a-pan-caspase inhibitor zVAD-fmk (25 µM). Treatment with TNF (10 ng/ml) + cycloheximide (CHX, 10 µg/ml) for 6 h was used as a control. EC death was determined by FITC-conjugated annexin-V and propidium iodide (PI) staining followed by FACS analyses. A. FACS for the zVAD groups are shown. The percentages of necroptotic cells (Upper left, UL) and apoptotic (Lower right, LR) are indicated. Similar data were observed in two additional independent experiments. B–C. The percentages of necroptotic cells and apoptotic are quantified in B (−zVAD) and C (+zVAD). All values were triplicate averages with standard deviations. **, P<0.05; *, P<0.01. D. Cell lysates were analysed for p-MLKL by Western blot with a phospho-specific antibody (p-Ser385). Actin was used as a control.

The isocyanate-generating agents strongly induce a non-apoptotic death in EC. Activation of ASK1-JNK signaling has been implicated in EC death. To determine the effects of the isocyanate-generating agents on EC death, BAEC were incubated with Laromustine, BCNU, 90CE, or 101MDCE, each at a concentration of 200 µM for 6 h. EC death was determined by FITC-conjugated annexin-V and propidium iodide (PI) staining followed by FACS analyses. Annexin-V binds to phosphatidylserine moieties exposed on an apoptotic but not necroptotic cell surface, whereas PI stains the DNA of necrotic and dead cells when their membranes are permeable. As a control, EC were also treated with TNF (10 ng/ml) in the presence of the protein synthesis inhibitor cycloheximide (CHX, 10 µg/ml), which is known to induce apoptotic death in EC. As shown in Fig. 5A with quantification in Fig. 5B, TNF+CHX induced a high level of apoptosis (27.8%) characterized by Annexin-V^+^PI^−^ staining. Laromustine, 101MDCE and BCNU did not induce apoptosis but strongly induced necroptosis characterized by Annexin-V^−^PI^+^ staining (36, 25.6 and 24.7%, respectively). 90CE, which lacks the carbamoylating activity of Laromustine, weakly induced EC necrosis (14%). Two major differences between apoptosis and necroptosis at molecular levels: 1) apoptosis is caspase-8-dependent; 2) in contrast, caspase-8 inhibits the assembly of necroptosome consisted of RIP1-RIP3-MLKL (mixed lineage kinase domain–like protein) where phosphorylation RIP3 and MLKL is critical step for activation of necroptosis [38]. Consistent with previous reports [39], a pan-caspase inhibitor zVAD-fmk drastically blocked TNF+CHX-induced apoptosis. in contrast, inhibition of caspase by zVAD-fmk augmented Laromustine, 101MDCE and BCNU-induced necroptosis (Fig. 5C). Moreover, Laromustine, 101MDCE or BCNU-induced phosphorylation of MLKL was enhanced by zVAD-fmk (Fig. 5D). These findings suggest that isocyanates induce EC death through a necroptotic pathway.

**Figure 6:**
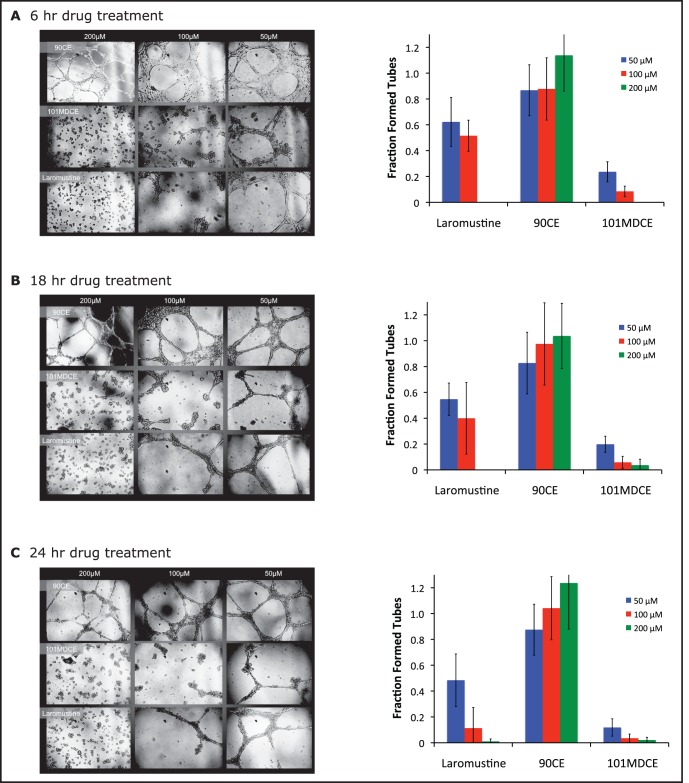
Inhibition of EC tube formation in vitro by 101MDCE. Human umbilical vein endothelial cells (HUVECs) were treated with 50 µM, 100 µM, or 200 µM Laromustine, 90CE, or 101MDCE (or DMSO control). 3×104 cells/cm2 were plated onto 96-well plates containing a thin film of Matrigel (BD Bioscience). The cells were examined at 6 hrs (A), 18 hrs (B), and 24 hrs (C) using a phase contrast inverted microscope. Tube structures were quantified by placing the microscope over the center of the well and counting fully formed tubes and partially formed tubes (defined as more than three-quarters closed) within view. Tube formation was normalized in each case to that of the DMSO control.

The isocyanate-generating agents inhibit EC tube formation in vitro. Angiogenesis, new blood vessel formation, is involved in tumor growth and metastases [40]. Since EC survival is critical for angiogenesis, we determined the effects of the isocyanate-generating agents on EC angiogenesis by an in vitro tube formation assay. EC were seeded on Matrigel in the presence of 50 µM of Laromustine, 101MDCE, 90CE or BCNU for 24 h. Tube formation was visualized microscopically and quantified by measuring tube area. The results showed that Laromustine, 101MDCE, and BCNU, but not 90CE, strongly inhibited EC tube formation (Fig. S1). We then analyzed the effects of Laromustine, 90CE, or 101MDCE (or DMSO control) on HUVECs tube formation at 50 µM, 100 µM, or 200 µM for various times. Our results showed that Laromustine and 101MDCE, but not 90CE, inhibited HUVEC tube formation in a dose and time-dependent manner. 101DMCE had the strongest inhibitory effect on tube formation (Fig. 6A–C). These findings directly correspond to the observed induction of ASK1-JNK signaling and EC death.

## Discussion

In the present study, we showed that 101MDCE, an analog of Laromustine that generates only carbamoylating activity and not a chloroethylating species, activated ASK1-JNK/p38 and non-apoptotic signaling in EC. The isocyanate-generating agents (Laromustine, 101MDCE, and BCNU) had no effect on the kinase activity of purified ASK1 or JNK or the catalytic activity of the ASK1 inhibitor Trx. However, the isocyanate-generating compounds significantly decreased the reductase activity of TrxR1, an enzyme that maintains Trx in a reduced active form. 90CE was a far less potent inhibitor of TrxR1 activity. Furthermore, 101MDCE dissociated ASK1 from Trx, but not from the phosphoserine-binding inhibitor 14-3-3, in both whole cells and cell extracts. These findings suggest that methyl isocyanate, which can carbamoylate free thiol groups of proteins, induced dissociation of ASK1 from Trx either directly by carbamoylating the critical Cys groups in the ASK1-Trx1 complex or indirectly by inhibiting TrxR1.

Methyl isocyanate generated from Laromustine is clearly an important component in the action of this antineoplastic agent. The carbamoylation of cysteine sulfhydryl groups in the active sites of key enzymes can be inhibitory and contribute to the function of methyl isocyanate. Of particular interest have been enzymes involved in DNA repair, given the synergistic cytotoxicity exhibited by the combination of methyl isocyanate and 90CE [2] and the increase in DNA cross-links produced by 90CE when combined with 101MDCE [1]. We have demonstrated that the direct repair protein AGT is a target of the carbamoylating activity of Laromustine [1], [3], [4]. Furthermore, DNA polymerization [41] and end-joining [42] activities are inhibited by the carbamoylating activity from 2-chloroethyl isocyanate of BCNU. Pre-clinical and clinical studies involving BCNU have also identified GR and TrxR as targets for 2-chloroethyl carbamoylation [43]. The carbamoylating activity of BCNU has often been considered a deleterious consequence of the drug's activation [44], while other studies have suggested that the isocyanate’s role is insignificant [45] or beneficial [42]. It is also clear that the alkyl group of the isocyanate species plays a role in its function. Other structurally distinct nitrosoureas yield different isocyanates whose reactivity and pharmacology differ greatly [46]. In addition, we have previously shown that methyl isocyanate from Laromustine and 101MDCE does not inhibit cellular GR, while 2-chloroethyl isocyanate from BCNU potently inhibits cellular GR [9]. In the present study, we have identified the ASK1/JNK-p38 signaling pathway as a new target of methyl isocyanate. We also demonstrate that like BCNU, Laromustine and 101MDCE are potent inhibitors of TrxR1. TrxR and Trx are highly expressed in malignant tissues, unlike GSH or GR [10]–[12], [47]. As such, TrxR is thought to be a potential target for antitumor therapy.

**Figure 7:**
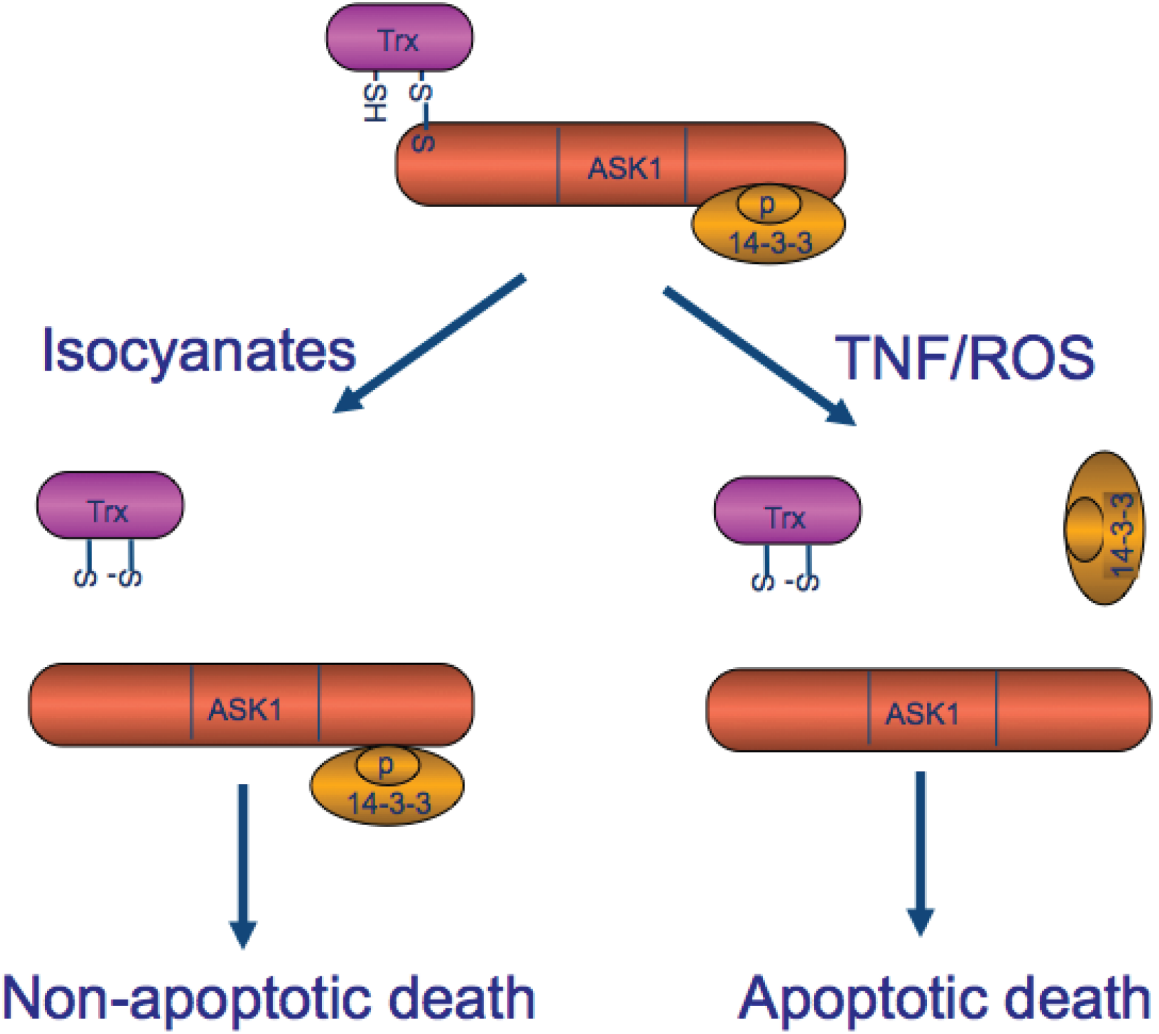
A model for Laromustine-derived isocyanate in activation of ASK1-dependent non-apoptotic cell death (necroptosis). In resting cells, ASK1 is maintained in an inactive state by two intracellular inhibitors Trx and 14-3-3. Trx-ASK1 associate through an intermolecular disulfide bond, while 14-3-3 binds to phosphoserine-967 on ASK1. Apoptotic stimuli (e.g., TNF +CHX and reactive oxygen species) induce dissociation of ASK1 from both Trx and 14-3-3, leading to ASK1 activation and caspase-dependent cell apoptosis. However, Laromustine-derived isocyanate dissociates ASK1 from Trx but not from 14-3-3, and induces a non-apoptotic EC death. This is likely a form of necroptosis, evident by phosphorylation of MLKL and promotion by pan-caspase inhibitors. SH: the thiol group from a cysteine residue of Trx or ASK1. S-S: the disulfide functional group forms an intermolecular (Trx-ASK1) or an intramolecular (Trx) interaction.

The most significant finding of our study is that 101MDCE dissociates ASK1 from its inhibitor Trx, but not from 14-3-3, leading to activation of ASK1-JNK signaling and EC necroptotic death (Fig. 7 for a model). The reduced form of Trx directly associates with ASK1 in the N-terminal domain of ASK1, inhibiting its kinase activity [13] and blocking activation of ASK1 by TNF [13], [14]. Neither the oxidized form (intramolecular disulfide between C32 and C35) nor the redox-inactive form (the double-mutation at catalytic sites C32 and C35) of Trx binds to ASK1. Death signals activate ASK1 in part by oxidizing Trx to release it from ASK1 [13], [14]. We have recently shown that a single Cys residue in the catalytic site of Trx (C32 or C35) and C250 of ASK1 are critical for the formation of the complex. Furthermore, Trx-C32S and Trx-C35S constitutively bind to ASK1, even in the presence of hydrogen peroxide in vitro or of TNF in vivo, most likely because they cannot be oxidized to form a disulfide bond between the two catalytic cysteines leading to its release from ASK1 [14], [16]. These results suggest that Trx is critical for the regulation of ASK1 activity. 101MDCE disrupts the ASK1-Trx complex in both whole cells and cell extracts. Moreover, 101MDCE dissociates ASK1 from Trx-C35 (not shown), which can no longer form intramolecular disulfide bonds. These findings suggest that the methyl isocyanate from 101MDCE may directly modify critical Cys residues either on ASK1 or Trx1.

Our findings suggest that methyl isocyanate dissociates ASK1 from Trx but not from 14-3-3 and leads to the non-apoptotic death of EC (Fig. 7). Previously, we have shown that apoptotic stimuli such as TNF together with protein synthesis inhibitor cycloheximide (CHX) specifically induce dissociation of ASK1 from both Trx and 14-3-3 leading to EC apoptosis [14]–[16], [27], [28], [48]. TNF induced reactive oixygen species (ROS) likely contribuyes to Trx1 release by oxidizing Trx1. TNF-activated AIP1 recruits PP2A, a phosphatase that dephosphorylates the 14-3-3 binding site pSer-967 on ASK1, to facilitate the 14-3-3 release [14]–[16], [27], [28], [48]. Although ASK1 has been reported to induce apoptosis by triggering a mitochondria-dependent pathway [21], ASK1 also induces non-apoptotic cell death [22]. Interestingly, the overexpression of the N-terminal domain of ASK1 is sufficient to induce non-apoptotic cell death, via a process involving the death protein Daxx [22]. It is conceivable that methyl isocyanate releases Trx1 from the N-terminal domain of ASK1 to facilitate formation of non-apoptotic complexes such as ASK1-Daxx without affecting AIP1-dependent dissociation of the ASK1-14-3- compolex and ASK1-dependent apoptotic pathways. Our data suggest that methyl isocyanate likely induces necroptotic death in EC. This is supported by the following data: 1) methyl isocyanate specifically induces phosphorylation of MLKL, a critical step for activation of necroptosomes; 2) methyl isocyanate-induced EC death is not blocked, but is promoted by a pan-caspase inhibitor zVAD [38] [39]. It needs to further investigate the crosstalk between ASK1 and known RIP1/RIP3/MLKL necroptosome.

All of the agents investigated in the present study are cytotoxic by design. It is therefore likely that other consequences of methyl isocyanate exposure are contributing to cell death. In fact, there is evidence that alkyl isocyanates from Laromustine and BCNU can actually induce apoptosis in Chinese hamster ovary cells and human leukemia cells [1]. At the same time, 2-chloroethyl isocyanate from BCNU has been shown to inhibit caspase activity and its associated apoptosis in HT58 cells [49]. In addition, the chloroethylating activity of Laromustine and BCNU will obviously ablate cell survival. These and other possibilities are under further investigation.

Activation of ASK1 leads to cell death in many cell types including tumor cells and EC. Thus, ASK1 is a central target of many cellular survival factors, including Raf-1, Akt, and hsp90 [29]–[31]. The present study further supports the concept that ASK1 can be a potentially promising therapeutic target for anticancer drugs. Moreover, isocyanate-generating agents induce inhibition of EC tube formation, a process involved in angiogenesis that is critical for tumor growth and metastases [40], [50]. These findings suggest that methyl isocyanate generated from Laromustine may contribute to the antineoplastic activity of the prodrug in part, by interfering with tumor angiogenesis.

## Materials and Methods

### Constructs

Trx1, 14-3-3 and ASK1 constructs were described previously [14], [26].

### Antibodies

A rabbit polyclonal antibody against Trx1 was generated by Cocalico Biologicals Inc. (Reamstown, PA) by immunizing rabbits with GST-Trx1. We obtained anti-ASK1 (H300), anti-14-3-3, and anti- IκBα antibodies from Santa Cruz Biotechnology (Santa Cruz, CA). Phospho-specific antibodies against p-ASK1, p-p38, p-Akt were purchased from Cell Signaling (Beverly, MA). Anti-p-JNK was from Biosource (Camarillo, CA) and anti-HA was from Roche (Indianapolis, IN). Phospho-MLKL (p-Ser385) was purchased from Abcam and zVAD-fmk was from Calbiochem.

### Cells, cytokines and inhibitors

Human umbilical vein endothelial cells (HUVECs) and bovine aortic ECs (BAECs) were purchased from Clonetics Corp. (San Diego, CA). Human umbilical vein EC (HUVECs) were cultured in modified M199 culture medium containing 20% (v/v) heat-inactivated bovine fetal calf serum (FCS), 100 µg/mL heparin sodium salt, 30 µg/ml endothelial cell growth supplement, 2 mM L-glutamine, 60 units/mL penicillin, and 0.5 µg/mL streptomycin at 37°C in 5% CO_2_ on gelatin-coated tissue culture dish. Cells were used at passages 2–4. Human recombinant TNF, used at 10 ng/mL, was from R&D Systems Inc. (Minneapolis, MN).

### Enzymes and biochemicals

Laromustine, 101MDCE, and 90CE were synthesized, purified, and characterized as described elsewhere [3], [4], [35], [51]. BCNU was purchased from Sigma (St. Louis, MO). Compounds were dissolved in dry DMSO to concentrations of 200 µM. Stock solution aliquots were stored desiccated at −20°C. Dilutions were also prepared in dry DMSO. Purified rat liver TrxR1, purified human recombinant Trx, and purified bovine insulin were obtained from Sigma. Purified human recombinant ASK1, human recombinant JNK1α1, bovine myelin basic protein, and biotinylated ATF2 peptide (aa 19–96) were obtained from Upstate (Waltham, MA). Streptavidin-coated membranes were purchased from Promega (Madison, WI). All other purchased reagents were of the highest quality.

### Immunoprecipitation and immunoblotting

HUVECs or BAECs after various treatments were washed twice with cold PBS and lysed in 1.5 mL of cold lysis buffer (50 mM Tris-HCl, pH 7.6, 150 mM NaCl, 0.1% Triton X-100, 0.75% Brij 96, 1 mM sodium orthovanadate, 1 mM sodium fluoride, 1 mM sodium pyrophosphate, 10 µg/mL aprotinin, 10 µg/mL leupeptin, 2 mM phenylmethylsulfonyl fluoride, and 1 mM EDTA) for 20 min on ice. Protein concentrations were determined with a Bio-Rad kit. For immunoprecipitations to analyze protein interactions in cells, 400 µg of cell lysate supernatant were pre-cleared by incubating 5 µg of normal rabbit serum and protein A/G agarose (Santa Cruz) on a rotator at 4°C overnight. The lysates were then incubated with 5 µg of the first protein-specific antiserum (e.g., anti-Trx1) for 2 h with 50 µL of protein A/G agarose. Immune complexes were collected after each immunoprecipitation by centrifugation at 14,000×g for 10 min followed by 4 washes with lysis buffer. The immune complexes were subjected to SDS-PAGE followed by immunoblotting (Immobilon P; Millipore, Milford, MA) with the second protein (e.g., ASK1-specific antibody; Santa Cruz Biotechnology Inc.). Chemiluminescence was detected using an ECL kit according to the instructions of the manufacturer (Amersham Life Science, Arlington Heights, IL).

### ASK1 and JNK kinase assays using cell lysates

ASK1 and JNK assays were performed as described previously [14], [26] using GST-MKK4 and GST-c-Jun (1–80) fusion proteins as substrates, respectively. Briefly, a total of 400 µg of cell lysate was immunoprecipitated with 5 µg of antibody against ASK1 or JNK1 (Santa Cruz). The immunoprecipitates were mixed with 10 µg of GST-MKK4 or GST-c-Jun (1–80) suspended in kinase buffer (20 mM Hepes, pH 7.6, 20 mM MgCl_2_, 25 mM β-glycerophosphate, 100 µM sodium orthovanadate, 2 mM DTT, 20 µM ATP) containing 1 µL (10 µCi) of [γ-^32^P] ATP. The kinase assay was performed at 25°C for 30 min. The reaction was terminated by the addition of Laemmli sample buffer and the products were resolved by 12% SDS-PAGE, followed by the transfer of protein to an Immobilon P membrane. The phosphorylated GST-MKK4 or GST-c-Jun (1–80) was visualized by autoradiography. The membrane was further used for Western blotting with anti-ASK1 or anti-JNK1.

### GST-Trx pull-down assay

The GST fusion protein preparation and GST pull-down assay were performed as described previously [14], [26]. Briefly, GST-Trx fusion proteins expressed in Escherichia coli XL-1 blue were affinity purified on glutathione-Sepharose beads. Four hundred µg of cell lysate expressing ASK1 was incubated overnight at 4°C with 10 µg of GST-Trx1 or GST-14-3-3 bound to glutathione-Sepharose in the lysis buffer in the presence of various agents. The beads were washed 4 times with the lysis buffer before the addition of boiling Laemmli sample buffer. Bound ASK1 proteins were resolved on SDS-PAGE and detected by Western blotting with anti-ASK1 for full-length ASK1 or anti-Flag antibody for Flag-tagged ASK1-N.

### ASK1 kinase assay using purified enzyme

The activity of purified ASK1 was assessed using myelin basic protein and ^32^P-labeled ATP as substrates in vitro as we described previously [52]. Labeled myelin basic protein products were quantified using phosphorimagery on SDS-PAGE separated reactions. One unit/mL of ASK1 was pre-incubated with 0, 50, 200, or 400 µM Laromustine, BCNU, 90CE, or 101MDCE for 1 h in 20 mM MOPS (pH 8), 1 mM EDTA, 0.01% Triton X-100, 5% glycerol, and 10% DMSO at 25°C. The pre-incubated kinase was added to a reaction of the following final composition: 0.25 µ/mL ASK1, 20 µM myelin basic protein, 100 µM ATP (including 1.0 Ci/mmol [γ^32^P] ATP), 20 mM MOPS (pH 7), 10 mM MgCl_2_, and 2.5% DMSO in a total volume of 20 µL. Reactions were incubated at 25°C for 20 min and stopped with an equal volume of 2x Laemmli SDS-PAGE sample buffer. Twenty µL of stopped reactions were separated on 15% acrylamide (37.5∶1 w/w acrylamide:bis-acrylamide) denaturing mini-gels. The gels were dried and exposed to a phosphorimager screen for at least 1 h. The phosphorylated products were quantified using the ImageQuant software (Molecular Dynamics, Piscataway, NJ). Data were normalized to the positive control (ASK1 pre-incubated with DMSO alone) and the negative control (without ASK1) and reported as triplicate averages of percent activity with standard deviations.

### JNK1 kinase assay using purified enzyme

The activity of purified JNK1 was assessed using a biotinylated peptide derived from ATF2 and ^32^P-labled ATP as substrates in vitro. Labeled products were immobilized on a streptavidin-coated membrane and separated from the free label by extensive washing. One unit/mL of JNK1 was pre-incubated with 0, 50, or 200 µM Laromustine, BCNU, 90CE, or 101MDCE for 1 h in 50 mM Tris-HCl (pH 8), 0.1 mM EGTA, 0.03% Triton X-100, and 10% DMSO at 25°C. The pre-incubated kinase was added to a reaction of the following final composition: 0.25 u/mL JNK1, 5 µM ATF2-biotin, 100 µM ATP (including 1.0 Ci/mmol [γ^32^P] ATP), 50 mM MOPS (pH 7.4), 10 mM MgCl_2_, 100 µM EGTA and 2.5% DMSO in a total volume of 20 µL. Reactions were incubated at 25°C for 20 min and stopped using 0.5 volume of 7.5 M guanidine-HCl. Five µL of the stopped reactions were applied to a streptavidin-coated membrane. Free label was washed from the membrane 4x with 200 mL of 2 M NaCl, then 4x with 200 mL of 2 M NaCl in 1% H_3_PO_4_, and finally 2x with 100 mL of deionized water. The membranes were then dried and exposed to a PhosphorImager screen for no less than 1 h. Bound, labeled substrates were quantified using the ImageQuant software (Molecular Dynamics). Data were normalized to the positive control (JNK1 pre-incubated with DMSO alone) and the negative control (without JNK1) and reported as triplicate averages of percent activity with standard deviations.

### In vitro assay purified thioredoxin reductase

TrxR activity was determined by monitoring the reduction of 5,5′-dithiobis(2-nitrobenzoic acid) (DTNB) as measured by the increase in absorbance at 405 nm. Five µ/mL TrxR was pre-incubated with 0–100 µM Laromustine, BCNU, 90CE, or 101MDCE in 40 mM Tris-HCl (pH 8), 100 µM NADPH, and 10% DMSO for 3 h at 25°C. The pre-incubated enzyme was added to a reaction of the following final composition: 0.5 unit/mL TrxR, 5 mM DTNB, 300 µM NADPH, 100 mM potassium phosphate (pH 7.4), 1 mM EDTA, and 1% DMSO. Reactions were carried out in wells of a UV-transparent, flat-bottomed 96-well plate in a total volume of 100 µL and incubated at 25°C, while continuously measuring absorbance of 405 nm light in a SpectraMax plate reader (Molecular Devices). Activity was measured as the rate of absorbance change during the first 1 min of reaction progress. All reactions were performed in triplicate. Data were normalized to the positive control (TrxR pre-incubated with DMSO) and the negative control (without TrxR) and expressed as percent activity. IC_50_ values were calculated by fitting the data to the following hyperbolic equation: 100/(1+(x/a)^b^), where ‘x’ refers to percent activity and ‘a’ is solved as the IC_50_ value.

### In vitro assay of purified thioredoxin

The ability of Trx to catalytically reduce biomolecules was assessed using an assay in which insulin and DTT were used as substrates in vitro [53]. The product of this reaction is insoluble at neutral pH and is measured by turbidity. Fifty µM Trx was pre-incubated with 0 or 100 µM Laromustine, BCNU, 90CE, or 101MDCE for 3 h in 50 mM Tris-HCl (pH 8) and 5% DMSO at 25°C. The pre-incubated enzyme was added to a reaction of the following final composition: 5 µM Trx, 200 µM insulin, 100 mM potassium phosphate (pH 7.4), 2 mM EDTA, and 1% DMSO. Reactions were initiated in wells of a clear, flat-bottom 96-well plate with DTT (final concentration = 330 µM) in a total volume of 100 µL. Reactions were incubated at 25°C while continuously measuring absorbance of 650 nm light in a SpectraMax plate reader (Molecular Devices). The empirical absorbances went unchanged for the first 1–4 min before rapidly increasing. Activity was measured as the rate of absorbance increase in the most linear 1-min segment of the initial reaction progress. Data are normalized to the positive control (Trx pre-incubated with DMSO alone) and the negative control (without Trx) and reported as triplicate averages of percent activity with standard deviations.

### Quantitation of apoptotic and non-apoptotic cell death

Cell killing assayed was performed as described previously with a modification [15], [26]–[28]. The PI-exclusion method for loss of integrity of cell membranes was used to assess viability. In brief, cells were suspended in PBS containing 25 µg/ml PI for 5 min at 37°C and then subjected to analytic flow cytometry on a FACSort (BD Biosciences) immediately after labeling. A light scatter gate was set up to eliminate cell debris from the analysis. The PI fluorescence signal was recorded on the FL3 channel and analyzed by using CellQuest software. Phosphatidylserine (PS) translocation, which precedes loss of PI exclusion in apoptotic cell death, was assessed by an annexin V-FITC staining kit (Roche) following the manufacturer’s protocol. For nuclear morphology, cells were stained with DAPI and apoptotic cell (nuclei condensation) were visualized under UV microscope.

### EC tube formation assay in Matrigel culture

Matrigel culture was performed as described previously [54]. Briefly, 10x RPMI 1640 medium (Gibco), neutralizing buffer (260 mmol/L NaHCO_3_, 200 mmol/L HEPES, 50 mmol/L NaOH), and Matrigel (Vitrogen, The Netherlands) were mixed at a ratio of 1∶1∶8 on ice, and then added to a 24-well plate (400 µL/well) and the plate was incubated at 37°C for 1 h. Human umbilical vein endothelial cells (HUVECs) were seeded on Matrigel at 2×10^4^/well and the plate was incubated overnight in 5% CO_2_ at 37°C. The medium was removed and cells were washed with 1× PBS gently. The Matrigel mixture prepared as above was added to cells that were already attached to the first layer of the Matrigel. The plates were incubated for 30 min at 37°C keeping the plate away from CO_2_. EC medium was added at 500 µl/well and the plates were incubated in 5% CO_2_ at 37°C. EC medium was changed every 2–3 days and tube formation was observed after 36–48 h. The branched EC network was visualized microscopically (4x) and images from three different areas in each well were captured using a digital camera and the area covered by branched cells (in mm^2^) was measured and analyzed using the NIH Image software.

### Statistical analysis

Data are presented as the means ±SD. For Western blots, kinase activity, apoptosis and reporter gene assays, experiments were performed at least twice with duplicates. Analyses of the densitometry were performed using NIH Image. Results were then normalized for comparison among different experimental groups by arbitrarily setting the value of control cells to 1.0. Statistical analyses were performed with StatView 4.0 package (ABACUS Concepts, Berkeley, CA). Differences were analyzed by an unpaired two-tailed Student t test. Values of p<0.05 were considered significant.

## Supporting Information

Figure S1 **Inhibition of EC tube formation in vitro by 101MDCE.** Human umbilical vein endothelial cells (HUVECs) were seeded on Matri-gel in the presence of 50 µM of Laromustine, 101MDCE, 90CE or BCNU in DMSO. DMSO was used as a control. A. 24 h later, Tube formation was stained for 30 minutes with 0.2% crystal violet in 10% ethanol, and visualized microscopically. Representative images from 5 fields are shown. Branch points as indicated by arrowheads. Scale bar: 100 µm. B. Tube formation was quantified by measuring branching points of the vascular network by taking the control (Ctrl) as 1.0. C. Fraction tube formation was normalized in each case to that of the control. Data are mean ± SEM from three independent experiments. **, P<0.05; *, P<0.01.
(TIF)
