# Supplementary figures and images for: Carbamoylating Activity Associated with the Activation of the Antitumor Agent Laromustine Inhibits Angiogenesis by Inducing ASK1-Dependent Endothelial Cell Death
Source: PLoS One. 2014 Jul 28;9(7):e103224. doi: 10.1371/journal.pone.0103224 (PMC4113355; doi:10.1371/journal.pone.0103224)

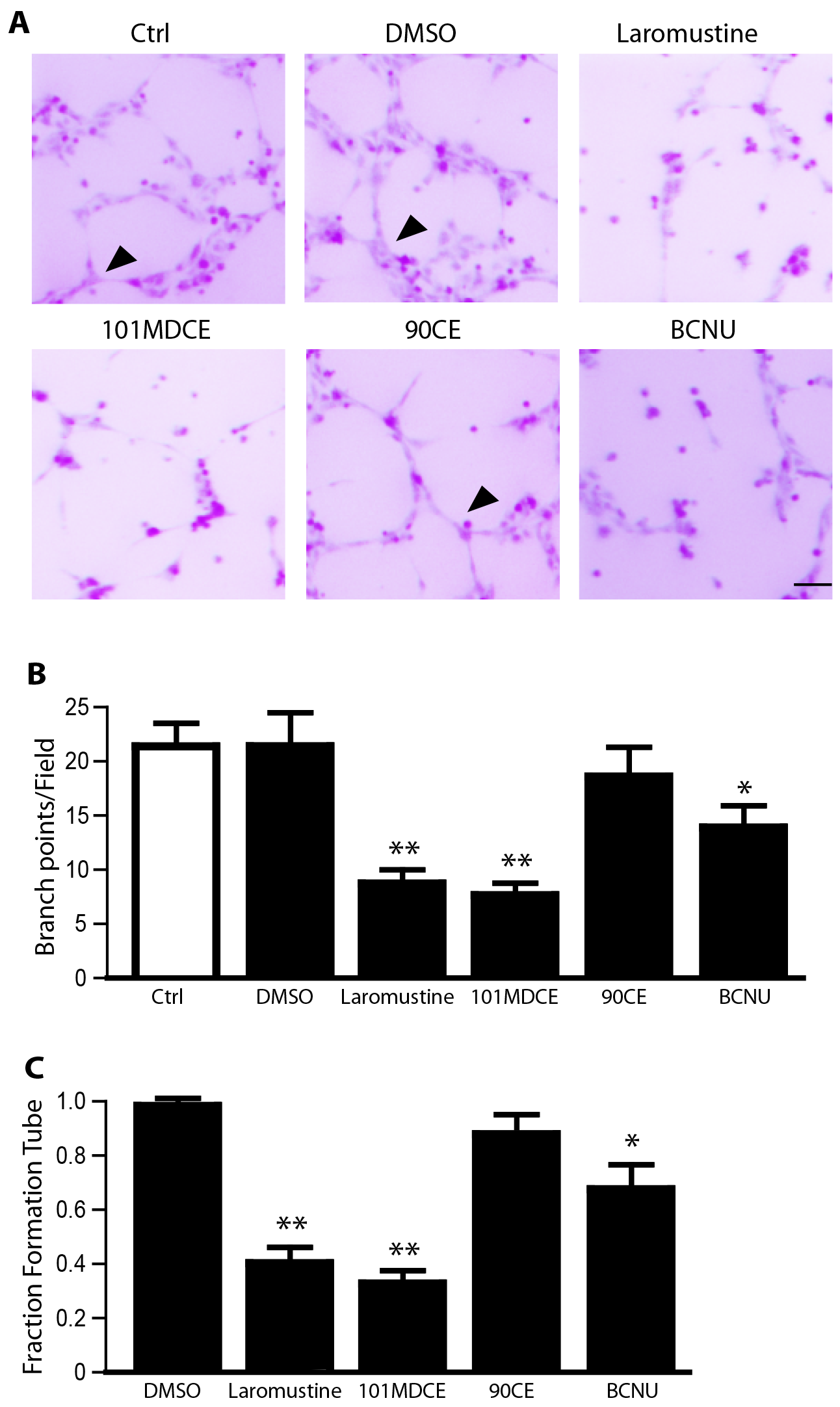

Supplement: Figure S1 — Inhibition of EC tube formation in vitro by 101MDCE. Human umbilical vein endothelial cells (HUVECs) were seeded on Matri-gel in the presence of 50 µM of Laromustine, 101MDCE, 90CE or BCNU in DMSO. DMSO was used as a control. A. 24 h later, Tube formation was stained for 30 minutes with 0.2% crystal violet in 10% ethanol, and visualized microscopically. Representative images from 5 fields are shown. Branch points as indicated by arrowheads. Scale bar: 100 µm. B. Tube formation was quantified by measuring branching points of the vascular network by taking the control (Ctrl) as 1.0. C. Fraction tube formation was normalized in each case to that of the control. Data are mean ± SEM from three independent experiments. **, P<0.05; *, P<0.01. (TIF) [file pone.0103224.s001.tif]
